# Impact of Job Satisfaction and Social Support on Job Performance Among Primary Care Providers in Northeast China: A Cross-Sectional Study

**DOI:** 10.3389/fpubh.2022.884955

**Published:** 2022-06-21

**Authors:** Di Liu, Xu Yang, Congyi Zhang, Wenlin Zhang, Qiaoran Tang, Yujin Xie, Lei Shi

**Affiliations:** ^1^School of Economics and Management, Harbin Engineering University, Harbin, China; ^2^School of Marxism, Harbin Medical University, Harbin, China; ^3^The Second Affiliated Hospital of Harbin Medical University, Harbin, China; ^4^Heilongjiang Institute of Standardization, Harbin, China; ^5^Children's Hospital of Soochow University, Suzhou, China; ^6^Beijing Rehabilitation Hospital, Capital Medical University, Beijing, China; ^7^School of Health Management, Southern Medical University, Guangzhou, China; ^8^Public Health Emergency and Health Education Base, Guangzhou, China

**Keywords:** primary care providers, job satisfaction, social support, job performance, China

## Abstract

**Background:**

Primary health care institutions face major challenges in maintaining the accessibility and affordability of health services. This requires primary care providers to change and improve their performance. Therefore, Study on the job performance is conducive to improve the quality of primary health care services and the sense of access of primary care providers.To understand the current status of job performance among primary care providers in Heilongjiang Province, China, and explore the impact of job satisfaction and social support on job performance, further to improve the job performance of primary care providers and ensure the stable development of primary health services.

**Methods:**

A stratified sampling method was adopted to select 1,500 primary care providers from seven cities in Heilongjiang Province, China, using the gross domestic product development level of each city as a basis. A questionnaire survey was conducted (effective response rate was 85.8%) by using sociodemographic factors, job satisfaction scale, social support scale and job performance scale. One-way ANOVA or independent sample *t*-test was used to analyze the differences of demographic factors on job performance. Pearson correlation analysis was used to measure relationship between job satisfaction, social support and job performance. Hierarchical linear regression was used to analyze the relevant influencing factors associated with job performance among primary care providers.

**Results:**

Among the primary care providers who participated in this survey, the mean job performance score was 22.189 (SD = 7.695). The job performance of primary care providers was positively correlated with job satisfaction (r=0.574, *p* < 0.001), and was also positively correlated with social support (r = 0.534, *p* < 0.001). Model 3 showed that job satisfaction (β = 0.299, *p* < 0.001) and social support (β = 0.149, p <0.001) are positive predictors of job performance, respectively. Moreover, the regression relationship explained that 37.6% for the variation of the dependent variable.

**Conclusions:**

The job performance of primary care providers in Heilongjiang province is relatively low. Job satisfaction and social support are the relevant factors affecting the job performance of primary care providers. It is necessary to provide assistance to primary care providers in terms of family, organization, society, policy, etc., to improve their job performance, and to better provide high-quality health services to the grassroots.

## Introduction

Medical and Health Services are related to the physical and mental health of hundreds of millions of people and the well-being of thousands of households, and it is also a major livelihood issue (1). Since the release of the “Opinions of the Central Committee of the Communist Party of China and the State Council on Deepening the Reform of the Medical and Health System” in 2009, the Chinese government has made great efforts to develop the medical and health industry, and significant progress has been made so far in this regard (1). Medical and health service systems covering urban and rural areas have taken shape (2). Disease prevention and control capabilities have been continuously enhanced, medical security coverage has gradually expanded, the level of health technology has rapidly improved, and the health of the people has improved significantly (3). The main health indicators of Chinese residents are at the forefront of developing countries (3).

In the past 10 years, the pace of equalization of basic public health services has accelerated, and the number of primary health care service institutions has increased sharply, such that every citizen has equal access to basic health care services (4). Primary medical service institutions undertakes chronic disease management, health education, and other functions (5). While they operate at full capacity, there is still a huge demand for primary health care services (Individuals and families in the community have universal access to basic health care, which should be obtained in a way that they can accept and fully participate, and the community and the country can bear the costs incurred) at the grassroots level (6). Primary care providers(including general practitioners, nurses, public health practitioners, pharmacists, etc.) may lack professional training and practical experience or have relatively low levels of education, making it difficult to make full use of limited resources (7). Thus, the contradiction between supply and demand is further magnified. Moreover, primary health care institutions have few opportunities for promotion, and the implementation of relevant welfare benefits is relatively lagging. This affects employees' enthusiasm to a certain extent, and the phenomenon of brain drain is common (8). Therefore, strengthening research on primary care providers and eliminating the negative effects of professional plateau is of great practical significance for promoting primary care providers' careers (9).

For primary care providers, good job performance not only provides people with high-quality nursing services, but also gives primary care providers a sense of accomplishment from their work, thereby providing the society with more high-quality health protection services (10). Therefore, timely and accurate assessment of the job performance of primary care providers, and effective measures to improve their job performance can achieve a win-win situation for all parties. In order to make forward-looking decisions and development policies conducive to the development of grassroots medical and health care, it is necessary to research the current status of and factors influencing the performance of primary care providers. Previous studies have shown that job performance is affected by many factors, including job satisfaction, social support, work environment, welfare policies, pressure, and burnout (11–15).

Job satisfaction can be defined as people's subjective feelings about their jobs and other aspects of their jobs (16). The operational efficiency and productivity of primary health services institutions are largely determined by the efficient and job performance of primary care providers (17). High job satisfaction can not only help medical staff improve their self-confidence and professional identity, promote them to strictly perform their duties and actively improve the relationship with colleagues, but also effectively reduce the mental stress brought by work, thus having a positive impact on job performance (18, 19). Al-Ahmadi also found that job satisfaction is one of the most significant factors affecting job performance in the nursing industry (8). Therefore, there is a positive prediction between job satisfaction and job performance, and the higher the job satisfaction, the higher the job performance (8, 15, 16). However, Vaculik et al.'s study found no significant relationship between job satisfaction and job performance (20). Some theories simultaneously show that employees' job performance and job satisfaction are supported by society as intermediaries (21).

Social support is described as the sum total of external support obtained by individuals through social connections, for example, individuals can perceive influences from society, family, environment, etc. If from outside influence is positive, can make the individual in its positive effects on the regulation of emotional state, form a strong sense of identity and professional sense of belonging, to a certain extent, reduce or alleviate the physical and mental fatigue, so as to put more effort in work and concentration, promote the improvement of performance (22). It is found that the higher the individual's sense of organizational support is, the more likely the individual is to show the sense of collective honor and organizational discipline, reduce absenteeism rate, demission rate, job burnout and other negative emotions, better integrate into the work atmosphere, improve job performance; The more support individuals receive in the family environment, the more likely they are to support other members in the organization, enhance their sense of trust in the organization, and promote the improvement of job performance (23). Previous studies have shown that perceived social support from colleagues improves the reported level of job performance (24). Therefore, there is a positive prediction between social support and job performance, and the higher the social support, the higher the job performance (11).

Platis believed that social support can give people a sense of subjectivity and pleasure, and is a good way to release work pressure and promote job satisfaction (8). When primary care providers through various channels to obtain from the organization, co-workers, family, friends, and so on various aspects support, can greatly improve the perception of social identity and self-identity, help to improve work skills, release the pressure of work and reduce turnover intention, forming positive incentives, and the resulting professional identity and passion into work, More conducive to the improvement of job satisfaction, and thus improve job performance (25).

In China, Shao et al. found that a considerable number of primary care providers do not receive adequate social support when faced with excessive workload (26). In addition, there is a lack of continuing education and promotion opportunities, poor working conditions, and stagnant wage levels for a long time. This has a greater negative impact on job satisfaction and job performance (27). An increasing number of studies have also shown a positive correlation between social support and job satisfaction (28–30). The more social support people receive, the higher their satisfaction will be (28–30).

However, are job satisfaction and social support related to the job performance of primary care providers in Heilongjiang Province? This study aims to provide a useful reference for relevant departments to carry out targeted improvement work by evaluating and analyzing the impact of job satisfaction and social support on the performance of primary care providers.

## Methods

### Participants and Procedures

From June to August 2019, a cross-sectional survey was conducted among primary care providers in 40 community health service stations/centers in seven prefectures and cities in Heilongjiang Province, China. A stratified sampling method was used to select 1,500 primary health service stations/centers from 40 communities in seven prefectures and cities based on the GDP level of prefecture-level cities in Heilongjiang Province and using the Heilongjiang Provincial Statistical Yearbook in 2019 as a benchmark (31). Primary care providers were the subjects of the survey. Prior to the formal investigation and data collection, we conducted uniform training for the investigators. One hundred primary care providers were simultaneously selected from five community health service centers in Harbin for the pre-survey. According to the pre-survey results and expert opinions, the scale items were appropriately modified to form the final questionnaire. The investigators communicated and coordinated with the community health service centers or stations, and distributed the questionnaires to each practitioner after obtaining the consent of the director of the community health service center or station. A total of 1,500 questionnaires were distributed, and 1,350 questionnaires were recovered, After excluding those with obvious errors, incorrect answers to polygraph questions, and incomplete answers, a total of 1,287 valid questionnaires were obtained, with an effective recovery rate of 85.8%.

Inclusion criteria of participants were: (1) staff who had been engaged in primary health services for 1 year or more; (2) informed consent to participate in this research; (3) did not affect the work of the research subjects. Exclusion criteria of participants were: (1) who had been on duty for 3 months or more due to accumulative study, advanced studies, sick leave, etc., within a year; (2) and security personnel, interns, and advanced students of community health service centers/ stations.

## Measurements

### Demographic Characteristics

This study used a self-compiled general demographic questionnaire. The demographic information included nine items, including gender (male, female), marital status (have a spouse, no spouse), age (≤ 25 years old, 26–35 years old, 36–45 years old, and ≥46 years old), educational background (high school degree or below, University college, University degree or above), working years (≤ 10 years, 11–20 years, and ≥20 years), technical title (senior, intermediate, primary, none), average monthly income (<2,000 CNY, 2,001–3,000 CNY, 3,001–4,000 CNY, 4,001–5,000 CNY, >5,000 CNY), compilation category (regular staff, contract staff, equal pay for equal work)(32, 33), and sleep quality (very satisfied, relatively satisfied, general, relatively dissatisfied, and very dissatisfied).

### Job Satisfaction Scale

Job satisfaction was measured using the short-form scale of the Minnesota Job Satisfaction Scale (MSQ) compiled by Weiss et al. and has 20 items (34–36). Each item was scored by Likert 5-level and the positive items are assigned according to (1-strongly disagree, 2-disagree, 3-general, 4-agree, 5- strongly agree), and the range ranges from 20 to 100 points. The higher the score, the greater the individual's satisfaction with the job. The Cronbach's α coefficient of the scale in this study was 0.970.

### Social Support Scale

The Social Support Rating Scale adopts the English version of the Social Support Rating Scale compiled by foreign scholars Caplan et al. (37) to evaluate social support. The Social Support Scale has a total of 16 items (37, 38), and is divided into four dimensions: leadership support, colleague support, family and friends support, and social relationship support (37, 38). Leadership support refers to an individual's personal experience of being respected, understood, and supported by the superiors or supervisors of the unit (37, 38); colleague support refers to the individual's help and support from the skills and experience of the colleagues around him, as well as group relations, etc. (37, 38). An important source of support is an individual's subjective experience, and support from family and friends means that an individual receives material and direct assistance from family and friends (37, 38). In the traditional Chinese concept, family and friends are important life circles in a person's social life, and an important manifestation of the individual's objective support, which refers to the support an individual obtains at the social level, social network, and participation in social activities (39). Each item was scored by Likert 5-level, and the positive items are assigned thus: 1 =strongly disagree, 2 = relatively not disagree, 3 = general, 4 = relatively agree, 5=strongly agree. The total social support score was calculated on a scale of 16 to 80 points, with the higher the score, the higher the social support the individual receives. The Cronbach's α coefficient of the scale in this study was 0.943.

### Job Performance Scale

The job performance scale was evaluated using the Chinese version of the Job Performance Scale compiled by Chinese scholars Cheng Zhengfang and Tang Jing (40). The job performance scale has 12 items, and is divided into three dimensions: job performance, duty-keeping, and compliance (40). Each item was scored by Likert 5-level, and the positive items are assigned as follows: 1 = strongly disagree, 2 = relatively disagree, 3 = general, 4 = comparatively agree and 5 = strongly agree. The job performance score ranges from 12 to 60 points, and the higher the score, the higher the degree of individual job performance. The Cronbach's α coefficient of the scale in this study was 0.866.

### Data Analysis

The study used IBM SPSS Statistics for Windows, version 20.0. Descriptive statistical analysis methods included numbers (N), percentages (%), and average and standard deviation of demographic variables (41). One-way ANOVA analysis or independent samples *t*-test was used to compare the job performance of primary health care providers with different sociodemographic characteristics. The post-test method was used to compare the differences of job performance between different sociodemographic characteristics groups Pearson's correlation analysis was used to test the relationship between job satisfaction, social support and job performance. Hierarchical linear regression analysis was used to explore the associated factors with job performance, including *F* value, R change (ΔR^2^), standardized regression coefficients β; each step of the regression model was reported. All the research variables were tested for multiplicity. Multivariate collinearity was diagnosed by Variance Inflation Factor (VIF). A two-sided test *p* < 0.05 indicates that the difference is statistically significant.

### Strengthening the Reporting of Observational Studies in Epidemiology Statement

We declared that the Strengthening the Reporting of Observational Studies in Epidemiology guidelines are followed in this study.

### Patient and Public Involvement

Neither the patients nor the public were involved in the development of the methodology for the current study. However, academic discussion with previous scholars and current status of primary care providers' job performance have jointly promoted the design and implementation of this study.

## Results

### Demographic Characteristics of the Respondents

The majority of primary care providers who participated in this survey were women (83.6%), aged between 26 and 35 years (38.1%), most of whom were spouses (68.5%), and their education was concentrated at a University degree or above (53.2%). The technical title was mostly the primary title (42.2%), the working life was ≤ 10 years (54.7%), the type of establishment was mostly the appointment system (37.5%), and the average monthly income was 2,000–3,000 Yuan (34.2%). 41.3% of respondents had general sleep quality. See Table 1 for further details.

**Table 1:** Demographic characteristics of the respondents(*N* = 1,287).

**Demographic variables**	***N***	**%**
**Gender**		
Male	211	16.4
Female	1,076	83.6
**Age group**		
≤ 25 years old	180	14.0
26–35 years old	490	38.1
36–45 years old	363	28.2
≥46 years old	254	19.7
**Marriage status**		
Have a spouse	882	68.5
No spouse	405	31.5
**Record of formal schooling**		
High school degree or below	173	13.4
University college	429	33.3
University degree or above	685	53.3
**Technical title**		
Senior	161	12.5
Intermediate	279	21.7
Primary	543	42.2
None	304	23.6
**Working fixed number of year**		
≤ 10 years	704	54.7
11–20 years	256	19.9
≥20 years	327	25.4
**Type of personnel post allocation**		
Regular staff	481	37.4
Contract staff	483	37.5
Equal pay for equal work	323	25.1
**Average monthly income**		
<2,000 CNY	127	9.9
2,001–3,000 CNY	440	34.2
3,001–4,000 CNY	331	25.7
4,001–5,000 CNY	215	16.7
>5,000 CNY	174	13.5
**Quality of sleep**		
Very satisfied	86	6.7
Relatively satisfied	142	11.0
General	531	41.3
Relatively dissatisfied	395	30.7
Very dissatisfied	133	10.3

### Respondents' Job Performance Average Results

The average job performance scores of the primary care providers in terms of age (*F*=2.856, *p* < 0.05), professional title (*F*=3.438, *p* < 0.05), compilation category (F=3.558, *p* < 0.05), and sleep quality (F=2.434, *p* < 0.05) of job performance average scores were significantly different. After *post-hoc* tests, there were significant differences in the mean value of job performance between the groups aged 36–45 and ≤ 25 years (mean difference =-1.723, *P*=0.014) and between the groups aged 36–45 and 26–35 years (mean difference = −1.217, *P* = 0.022); there was no significant differences in the mean value of job performance between the groups aged 36–45 and ≥46 years (mean difference = −0.431, *P* = 0.492). There were significant differences in the mean value of job performance between the senior group and three other groups including intermediate group (mean difference = −2.043, *P*=0.007), primary group (mean difference = −1.402, *P* = 0.042) and none group (mean difference = −2.231, *P* = 0.003).There were significant differences in the mean value of job performance between very dissatisfied group and three other groups including very satisfied group (mean difference = −2.344, *P* = 0.028), general group (mean difference = −2.099, *P* = 0.005), and relatively dissatisfied group (mean difference = −1.914, *P* = 0.013); there was no significant differences in the mean value of job performance between the very dissatisfied group and the relatively satisfied group (mean difference = −1.091, *P* = 0.239). There were significant differences in the mean value of job performance between the equal pay for equal group and regular staff group (mean difference = 1.221, *P* = 0.027) and between the equal pay for equal group and contract staff group (mean difference = 1.391, *P* = 0.012). See Table 2 for further.

**Table 2:** Average job performance of respondents.

**Demographic variables**		**N**	**%**	**Mean**	**SD**	***F/t***	***p***
**Gender**						0.344	0.731
	Male	211	16.4	22.356	7.961		
	Female	1,076	83.6	22.156	7.646		
**Age group**						2.856	0.036
	≤ 25 years old	180	14.0	23.122	8.048		
	26–35 years old	490	38.1	22.616	7.752		
	36–45 years old	363	28.2	21.399	7.592		
	≥46 years old	254	19.7	21.831	7.387		
**Marriage status**						1.198	0.231
	Have a spouse	882	68.5	22.015	7.322		
	No spouse	405	31.5	22.568	8.449		
**Record of formal schooling**						0.172	0.842
	High school degree or below	173	13.4	22.353	8.013		
	University college	429	33.3	22.310	7.611		
	University degree or above	685	53.3	22.072	7.676		
**Technical title**						3.438	0.016
	Senior	161	12.5	20.627	6.609		
	Intermediate	279	21.7	22.670	7.078		
	Primary	543	42.2	22.030	7.919		
	None	304	23.6	22.859	8.253		
**Working fixed number of year**						2.059	0.128
	≤ 10 years	704	54.7	22.546	8.136		
	11–20 years	256	19.9	22.441	7.031		
	≥20 years	327	25.4	22.006	7.172		
**Type of personnel post allocation**						3.558	0.029
	Regular staff	481	37.4	21.946	7.633		
	Contract staff	483	37.5	21.776	7.162		
	Equal pay for equal	323	25.1	23.167	8.459		
**Average monthly income**						1.161	0.326
	<2,000 CNY	127	9.9	22.559	9.251		
	2,001–3,000 CNY	440	34.2	22.461	7.536		
	3,001–4,000 CNY	331	25.7	22.344	8.013		
	4,001–5,000 CNY	215	16.7	22.070	7.021		
	>5,000 CNY	174	13.5	21.081	6.778		
**Quality of sleep**						2.434	0.046
	Very satisfied	86	6.7	22.802	10.925		
	Relatively satisfied	142	11.0	21.549	6.272		
	General	531	41.3	22.557	7.391		
	Relatively dissatisfied	395	30.7	22.372	7.971		
	Very dissatisfied	133	10.3	20.459	6.684		

### Correlation Between Job Satisfaction, Social Support, and Job Performance

Table 3 shows the correlation between job satisfaction, social support, and job performance of primary care providers. The score of primary care providers' job performance was 22.189 (SD = 7.695). The job performance of primary care providers was positively correlated with job satisfaction (*r* = 0.574, *p* < 0.001), and was also positively correlated with social support (*r* = 0.534, *p* < 0.001). See Table 3 for further details.

**Table 3:** Pearson correlation among job satisfaction, social support and job performance.

**Variables**	**Mean**	**SD**	**1**	**2**	**3**
1. Job satisfaction	37.587	15.001	1		
2. Social support	33.036	12.851	0.745**	1	
3. Job performance	22.189	7.695	0.574**	0.534**	1

**p < 0.001 (two-tailed).

### Hierarchical Multiple Regression Analysis of Job Performance

The results of the hierarchical linear regression analysis of factors related to job performance are presented in Table 4. The collinearity diagnosis VIF is 3.033, far <10, indicating that there is no collinearity. Variables that are statistically significantly related to job performance in univariate analysis were used as control variables. As shown in Model 2, job satisfaction can positively predict job performance (β = 0.299, *p* < 0.001), and it can explain 32.4% of the variation in regression relationship. As shown in Model 3, social support can positively predict job performance (β = 0.149, *p* < 0.001), and it can explain 2.7% of the variation in regression relationship. See Table 4 for further details.

**Table 4:** Hierarchical linear regression for exploring the correlates of job performance.

**Variables**	**Model 1 (β)**	**Model 2 (β)**	**Model 3 (β)**
Age group			
26–35 years old	−0.478	0.185	0.272
36–45 years old	−1.790*	−0.561	−0.546
≥46 years old	−0.978	−0.007	0.195
Technical title			
Senior	−1.796*	−2.889**	−3.057**
Intermediate	0.361	−1.004	−0.874
Primary	−0.719	−1.085*	−0.879
Type of personnel post allocation			
Regular staff	0.714	0.078	−0.022
Equal pay for equal	1.210*	0.476	0.420
Quality of sleep			
Very satisfied	2.471*	0.210	0.379
Relatively satisfied	1.063	−1.030	−0.964
General	1.993*	−0.399	−0.170
Relatively dissatisfied	2.007*	0.507	0.600
Job satisfaction		0.299**	0.204**
Social support			0.149**
*F*	2.764*	52.432**	54.672**
*R^2^*	0.025	0.349	0.376
*Δ*R*^2^*	0.025	0.324	0.027

*p < 0.05, **p < 0.01.

## Discussion

This survey is a study of the current status of job performance among primary care providers in Heilongjiang Province, and explore the impact of job satisfaction and social support on job performance. The results showed that the job performance score of primary care providers was 22.189 (SD = 7.695), which was consistent with the research results of other scholars with low job performance (42). This phenomenon may be related to regional economic development, insufficient staffing of primary health service agencies, excessive individual workload, imperfect vacation system, imperfect specific responsibilities and requirements of employees, imperfect attendance system and reward and punishment system (43–45).

### Associated Factors With the Job Performance Among Primary Health Care Providers

In this survey, primary care providers of 36–45 years old were most likely to improve their job performance. They have a wealth of knowledge and experience (10, 46) that can provide young primary care providers with additional skills and experience. Primary care providers of 36–45 years old, most of whom are married and have children, and this may have something to do with the unique cultural background of China (47). In China, family relationship is the closest, warmest and strongest relationship, occupying the highest position in the main interpersonal relationship. Primary care providers (36–45 years old) face more pressure from their families, such as supporting parents, taking care of their spouses and bringing up children, than other age groups. Under the condition of equal energy, it may affect the energy put into work, and then lead to the decrease of job performance (48).

The results showed that equal pay for equal can significantly improve the job performance of primary care providers. Primary care providers who work in the same capacity tend to be more stressed and have higher workloads than regular staff and contract staff. Therefore, they need to strive for better development and higher income through hard work (49). The results also indicated that primary care providers with senior professional title can significantly improve their job performance. This may be due to the fact that primary care providers with senior technical title have achieved or partially achieved career planning at the occupation and income aspects, resulting in reduced work initiative and enthusiasm, thus affecting job performance (50, 51). Moreover, results also showed that job performance of primary care providers with very unsatisfactory sleep quality could be significantly improved. Long-term low-quality sleep will have a certain impact on the health of primary care providers, mainly manifested as low job performance, lack of concentration, it is difficult to ensure high job performance.

Hierarchical linear regression analysis showed that social support was positively correlated with job performance. Previous studies have shown that reducing social support can lead to the accumulation of potential stressors (52). Primary care providers can obtain social support from social activities and fully understand their roles in society (53). Primary care providers can also receive encouragement from social activities and help each other to enhance their sense of identity. More importantly, primary care providers can obtain social support from their daily contact (54). They can share experiences, avoid anxiety and depression, find more opportunities from social competition, and gain acceptance from others. The supportive working environment of community health service agencies can relieve tension and help cope with stressors, benefit the physical and mental health development of primary care providers, and better provide high-quality primary care services to others (55, 56).

Hierarchical linear regression analysis also showed that job satisfaction can positively predict job performance. Job satisfaction is a barometer that reflects the emotional status of primary care providers, playing an important role in the development of community basic health care (24, 57). Yet, policymakers and governments in the health system often ignore this key issue. Individual's attitude determines their behavior. High job satisfaction can effectively stimulate individual's work willingness and enthusiasm, invest more energy, and improve job performance. Effective incentive factors, such as the evaluation of personal honor, scientific and reasonable reward system and other measures can also effectively improve job performance. The relationship between job satisfaction and motivating factors needs to be met to improve job performance.

### Recommendations of the Research for the Primary Health Service

These results show that social support and job satisfaction are closely related to primary care providers' job performance. We also found that there is significant room for improving the job satisfaction of primary care providers. We, therefore, recommend that decision-makers provide more social support to primary care providers to reduce stress and promote physical and mental health. In addition, the government needs to recognize the imbalance in economic and social development among different provinces in China and provide more resources and subsidies to lacking regions. Third, managers should pay more attention to primary care providers with senior professional titles. Their work and life pressure is higher, give them more support and care, provide more opportunities for development and promotion, release more internal motivation. Fourth, health system reforms need to consider the potential side effects of some established policies on specific primary care providers. When demanders benefit from reforms, they may be indirectly affected (58).

## Limitations

This study has certain limitations. First, it was limited by personnel, time, and economic costs. The study adopted cross-sectional research methods, and did not conduct follow-up surveys of primary care providers. Second, the stratified sampling method was investigated in Heilongjiang Province, China. Third, the use of cross-sectional surveys may limit our ability to identify the causal relationship between job satisfaction, social support, and job performance.

## Recommendations for Future Research

The future of our research assumptions. First, a follow-up survey was conducted on primary care providers. Second, the scope of the investigation has been expanded and the investigation has been carried out nationwide. Thirdly, through longitudinal research, the relationship among job performance, job satisfaction and social support should be better studied. Fourthly, the time of this research is before the COVID-19. After the epidemic of COVID-19, we will carry out investigations to study their job performance.

## Conclusions

The job performance of primary care providers in Heilongjiang Province, China, is low. Professional titles, job satisfaction, and social support are the main factors that affect the performance of primary care providers. The importance of primary care providers' job performance is becoming increasingly prominent. To improve the job performance of primary care providers, it is necessary for the family, society, and country levels to form a joint force to establish and improve such job performance based on the work characteristics of the grassroots health service. Training programmes and unblocked career channels can encourage primary care providers to provide high-quality health services to the public, and also enhance the happiness and sense of acquisition of primary care providers, thus achieving a win-win situation, further developing grassroots health services, and providing development planning and reference opinions.

## Data Availability Statement

The datasets and/or analyses from the current study will be available from the corresponding authors upon reasonable request.

## Ethics Statement

Ethical approval to conduct this study was granted by the Research Ethics Committee of Children's Hospital of Soochow University (No. 2020CS054). The patients/participants provided their written informed consent to participate in this study.

## Author Contributions

DL, YX, and LS designed the study and revised the manuscript. XY, CZ, WZ, and QT collected data. XY, CZ, and QT analyzed the data. DL and LS drafted the manuscript. All authors reviewed the manuscript.

## Funding

This work was funded by the National Nature Science Foundation of China (72104098), Guangdong Basic and Applied Basic Research Foundation (2020A1515110369), Project funded by China Postdoctoral Science Foundation (2021M701592), and the Directive Project of Medical Scientific Research Foundation in Guangdong (A2022379). The funder had no role in the design and conduct of the study, collection, management, analysis, and interpretation of the data, preparation, review, or approval of the manuscript, and decision to submit the manuscript for publication.

## Conflict of Interest

The authors declare that the research was conducted in the absence of any commercial or financial relationships that could be construed as a potential conflict of interest.

## Publisher's Note

All claims expressed in this article are solely those of the authors and do not necessarily represent those of their affiliated organizations, or those of the publisher, the editors and the reviewers. Any product that may be evaluated in this article, or claim that may be made by its manufacturer, is not guaranteed or endorsed by the publisher.

## Abbreviations

GDP: Gross Domestic Product
COVID-19: 2019 novel coronavirus disease.
